# p25alpha Domain-Containing Proteins of Apicomplexans and Related Taxa

**DOI:** 10.3390/microorganisms11061528

**Published:** 2023-06-08

**Authors:** Ferenc Orosz

**Affiliations:** Institute of Enzymology, Research Centre for Natural Sciences, 1117 Budapest, Hungary; orosz.ferenc@ttk.hu

**Keywords:** apicortin, TPPP, Myzozoa, chrompodellids, dinoflagellates, perkinsids

## Abstract

TPPP (tubulin polymerization promoting protein)-like proteins contain one or more p25alpha (Pfam05517) domains. TPPP-like proteins occur in different types as determined by their length (e.g., long-, short-, truncated-, and fungal-type TPPP) and include the protein apicortin, which possesses another domain, doublecortin (DCX, Pfam 03607). These various TPPP-like proteins are found in various phylogenomic groups. In particular, short-type TPPPs and apicortin are well-represented in the Myzozoa, which include apicomplexans and related taxa, chrompodellids, dinoflagellates, and perkinsids. The long-, truncated-, and fungal-type TPPPs are not found in the myzozoans. Apicortins are found in all apicomplexans except one piroplasmid species, present in several other myzozoans, and seem to be correlated with the conoid and apical complex. Short-type TPPPs are predominantly found in myzozoans that have flagella, suggesting a role in flagellum assembly or structure.

## 1. Introduction

**Figure 1:**
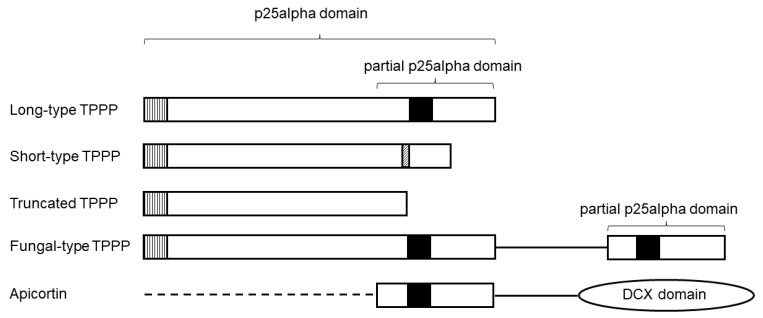
Schematic structure of some TPPP-like proteins. Highly conservative sequence motives are denoted with black boxes (GxGxGxxGR), vertical-striped boxes (L(V)xxxF(Y)xxF), and diagonal-striped boxes (GGP). The dashed line represents a disordered region unique to some apicortins.

TPPP-like proteins contain one or more p25alpha domains [1]. They obtained their name after tubulin polymerization promoting protein (TPPP1), the first identified member of this protein family [2,3]. Originally, it was named p25alpha protein, which became the eponym of the domain [4]. P25alpha (Pfam05517; IPR008907) is not a structural domain but is derived from a sequence alignment (https://bioinf.umbc.edu/DMDM/generatelogo.php?accession=pfam05517 (accessed on 5 March 2023)). The p25alpha domain is exclusive to eukaryotes and there is a strong correlation between its presence and the presence of the eukaryotic flagellum/cilium [5,6]. TPPP-like proteins occur in different types, such as long-, short- and truncated (the C-terminal third is completely missing) TPPPs, depending on the length of the p25alpha domain (about 160, 140, and 120 amino acids, respectively) (Figure 1). The main difference between long- and short-type TPPPs is that the C-terminal end of short-type TPPPs is incomplete; long-type TPPPs but not short-type ones contain a very conservative sequence of 31–32 amino acids, the most typical part of which is the GXGXGXXGR ‘Rossmann-like’ motif (Figure 1). Some TPPP-like proteins also possess another domain/region such as EF-hand (CDD:428504) or doublecortin (DCX; Pfam 03607, IPR003533) in addition to the p25alpha [1]. The latter one is named apicortin, which unifies partial p25alpha and DCX domains [7] (Figure 1).

The first TPPP-like protein, TPPP/p25 or TPPP1, was identified in mammalian brain [2,3,4] and its physiological significance is connected to the nervous system [8,9] as well as having a role in neurodegenerative disorders, as Parkinson’s disease and multiple system atrophy [10,11,12]. Obviously, the role of TPPP-like proteins will be different in eukaryotic microbes without a nervous system. However, their interaction with tubulin and the microtubular system is a conserved property [13,14,15].

**Figure 2:**
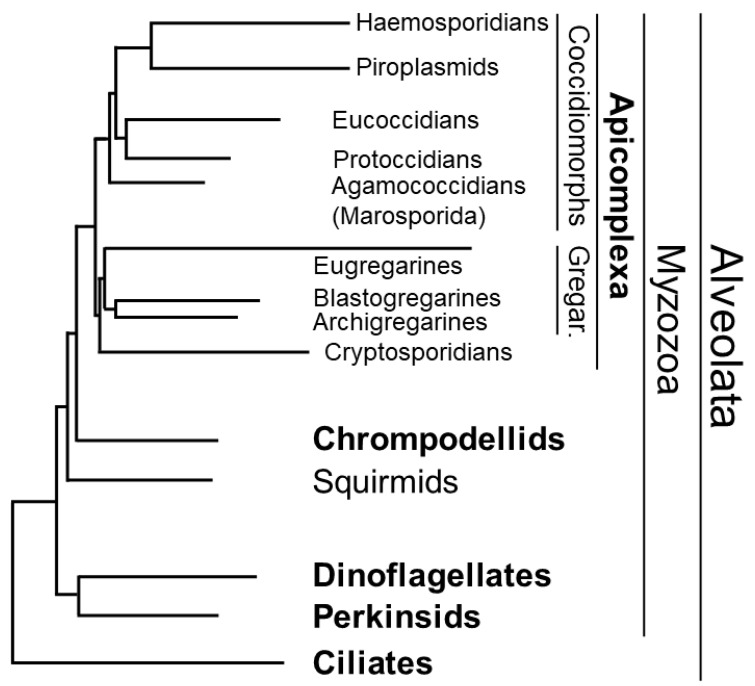
Phylogenetic tree of Alveolata based on Ref. [18]. Phyla are indicated by bold letters. Gregar. stands for gregarines.

The various members of the TPPP-like protein family are characteristic for the phylogenomic supergroups [1]. For example, animals contain only long-type TPPPs, except for the placozoan *Trichoplax adhaerens*, which contains apicortin instead [7]. Truncated- and fungal-type TPPPs occur only in Endopterygota (Holometabola) [16] and in fungi [6], respectively. In this article, the occurrence, and the possible role of TPPP-like proteins in Myzozoa, which include apicomplexans and related taxa, chrompodellids (chromerids plus colpodellids), dinoflagellates, and perkinsids, is reviewed (Figure 2). Myzozoans are a monophyletic clade, and a sister clade to the Ciliata, within Alveolata [17,18]. All myzozoans evolved from an ancestral myzocytotic feeder that possessed plastids, which predate the emergence of the Alveolata [19,20,21]. For the classification of Myzozoa, I accept what was used in Ref. [18] (Figure 2).

## 2. Methods

Accession numbers of protein and nucleotide sequences refer to the National Center for Biotechnology Information (NCBI) GenBank database unless otherwise stated. NCBI Blast was used to search databases [22] (http://www.ncbi.nlm.nih.gov/BLAST/). Whole sequences of various p25alpha domain-containing proteins were used as queries against protein and nucleotide (including transcriptome shotgun assemblies—TSAs; whole genome shotguns—WGSs; and expressed sequenced tags—ESTs) databases to find similar sequences in Myzozoa using BLASTP and TBLASTN analyses, respectively. The queries were *Tetrahymena thermophila* XP_001023601, *Plasmodium falciparum* XP_001350760, *Babesia bovis* XP_001610770, and *Trypanosoma brucei* XP_844424 for short-type TPPPs; *Drosophila melanogaster* NP_648881, *Caenorhabditis elegans* NP_491219, *Amphimedon queenslandica* XP_003384590, and *Monosiga brevicollis* XP_001743131 for long-type TPPPs; *Spizellomyces punctatus* XP_016604112, *Chytriomyces confervae* TPX65513, *Batrachochytrium dendrobatidis* XP_006680205, and *Allomyces macrogynus* KNE68590 for fungal-type TPPPs; *D. melanogaster* NP_001097567, *Danaus plexippus* XP_032527880, and *Nasonia vitripennis* XP_001604263 for truncated-type TPPPs; and *T. adhaerens* XP_002111209, *B. bovis* XP_001609847, *P. falciparum* XP_001351735, *Jimgerdemannia flammicorona* RUS30044.1, and *S. punctatus* XP_016606225.1 for apicortins. In addition, VEuPathDB resources [23] were also searched.

Multiple alignments of sequences were conducted by the Clustal Omega program [24]. The N-terminal amino acids before the p25alpha domain were trimmed. Bayesian analysis, using MrBayes v3.1.2 [25], was performed to construct phylogenetic trees. Default priors and the WAG [26] or GTR [27] models were used, assuming equal rates across sites. Two independent analyses were run with three heated and one cold chain (temperature parameter 0.2) for generations, as indicated in figure legends, with a sampling frequency of 0.01, and the first 25% of generations were discarded as burn-in. The two runs were convergent.

## 3. TPPP-like Proteins in Myzozoa

### 3.1. Apicortin

Apicortins unite two conserved domains, a DCX motif and a partial p25alpha sequence, that are separately found in other proteins, in doublecortins and TPPPs, respectively [7] (Figure 1). The DCX domain is named after the brain-specific X-linked gene doublecortin [28]. Both the p25alpha and the DCX domains play an important role in the stabilization of microtubules [2,3,28,29]; thus, a similar role was suggested for apicortin [7].

Apicortin was originally thought to occur only in apicomplexans and in the placozoan animal *T. adhaerens* [7]. Later, it was found that its occurrence is broader than thought earlier: it is present in chromerids (*Chromera velia* and *Vitrella brassicaformis*) [30] and in flagellated fungi [6,31,32]. The presence of apicortin in chromerids was not surprising, given the phylogenetic proximity and the structural similarity of chromerids and apicomplexans. (When apicortin was first identified in 2009 [7], chromerids were just discovered [33,34], and their genomes were sequenced a few years later [35].)

BLASTP analyses [22] were performed on myzozoan protein and nucleotide sequences available at the NCBI webpage using the sequences mentioned in Section 2 as queries. Since apicortins contain two different domains, various domain databases were also checked for proteins with both the DCX and the partial p25alpha domains that BLAST may not have been able to detect. The results of the search, i.e., the new apicortins not known before, are listed in Table 1. They were found in all myzozoan phyla, in Apicomplexa, chrompodellids, dinoflagellates, and Perkinsozoa. The newly re-interpreted [36,37] squirmids [38] (*Digyalum oweni* [39]) also possess it (the list of apicortins identified earlier is published in the references [6,7,31]).

Apicortin still seems to be a characteristic protein of apicomplexans; it occurs in all but one apicomplexan genome fully sequenced so far; the exception is the apicomplexan, with the smallest genome, *Babesia microti* [40]. In previous years, mainly medically important genera (e.g., *Plasmodium*, *Toxoplasma*, etc.) were sequenced; however, in recent years, transcriptomes and genomes of an increasing number of apicomplexans have been partially or completely established. They include gregarines as *Ancora sagittata*, *Cephaloidophora* cf. *communis*, *Polyrhabdina* sp., *Siedleckia nematoides*, *Selenidium pygospionis* [20], and *Porospora gigantea* [41], as well as coccidiomorphs as *Cardiosporidium cionae* [42], *Eleutheroschizon duboscqi*, *Rhytidocystis* sp. [20], and *Nephromyces* ex *Molgula occidentalis* [43]. All species listed here contain apicortin.

The photosynthetic chromerids, *C. velia* and *V. brassicaformis*, belong to a monophyletic group, called the chrompodellids, with heterotrophic colpodellids, namely, *Alphamonas edax*, *Voromonas pontica*, and *Colpodella angusta* [17,18]. It is clear that although apicortin has not been known in them until now, it was worth examining them in this regard (cf. Table 1). There are another two myzozoan phyla, Dinoflagellata and Perkinsozoa, which are sisters to each other, and together, are sisters to Apicomplexa and chrompodellids (Figure 2). So far, apicortin has not been detected in dinoflagellates, while there were traces of its presence in perkinsids [44,45]. For evolutionary reasons, it is obvious that if, on the one hand, apicomplexans and chromerids contain apicortins, and on the other hand, we can count on their presence in perkinsids, then their occurrence in dinoflagellates is not surprising (Table 1).

A few sequences are incomplete; this may be due to the fact that in the case of some species, a significant percentage of the genome/transcriptome data are missing, e.g., 48% of *Lankesteria abbotti* data are missing [20], and the N-terminus of the HBHB01002866 (Table 1) is not present in the available TSA sequence. The missing transcriptome data may also explain that while apicortin was identifiable in *C. angusta*, it was not found in the closely related (same family) *V. pontica*, in which 22% of the data are missing [20].

### 3.2. Short-Type TPPP

Short-type TPPPs have not been previously systematically investigated. They occur in various protists, mostly in Ciliata, Euglenozoa, and Chlorophyta [1], and they are present in Apicomlexa and Perkinsozoa [7]. Based on BLAST search (cf. Section 2), new short-type TPPPs were found in many myzozoans, not only in Apicomplexa and Perkinsozoa, but also in chrompodellids and dinoflagellates (Table 2). Within the apicomplexans, short-type TPPPs were found in Haemosporida, Piroplasmida, Eimeriidae, and Sarcocystidae, but they were absent in Cryptosporidiidae [7]. This analysis showed that they also occur in Nephromycida, gregarines (Eu-, Blasto-, and Archigregarinorida) and the recently defined class, Marosporida [36]. There are some characteristic sequences in short-type TPPPs. For example, one of the sequences, which is common with long-type TPPPs, is the L(V)xxxF(Y)xxF at the very beginning of the p25alpha domain. Another one is a GGP sequence in the C-terminal half of the protein (Figure 1 and Figure S1). The length of the proteins varies between 120 and 170 amino acids.

### 3.3. Multidomain Proteins Containing Short p25alpha Domains

As expected, the BLAST search found no truncated-, long-, or fungal-type TPPP-like proteins in myzozoan species. These TPPP-like proteins are specific for Endopterygota, Opisthokonta, and fungi, respectively. However, several multidomain proteins have been identified that contain two short-type p25alpha domains in addition to others (Table 3).

These proteins do not occur in apicomplexan species—not even as TSA or WGS sequences—but can be found in chrompodellids, dinoflagellates, and perkinsids. In chrompodellids, such a protein cannot be found in colpodellids but in chromerids, both in *C. velia* and in *V. brassicaformis.* The short-type p25alpha domain is present in duplicate in these proteins, which can be divided into three groups. In some cases, they do not even contain another domain (*C. velia*, *D. oweni*, *Symbiodinium* genus; *Perkinsus* genus). Strictly speaking, only these proteins can be considered to be TPPP-like. In other cases, an EF-hand or ankyrin repeats are present. In the third case, the duplicated p25alpha domains are associated with various catalytic domains, most often with a Nucleotidyl_ cyc_III one, which is typical of adenylate and guanylate cyclases; these are mostly proteins containing more than a thousand amino acids.

## 4. Phylogenetics

### 4.1. Apicortin

**Figure 3:**
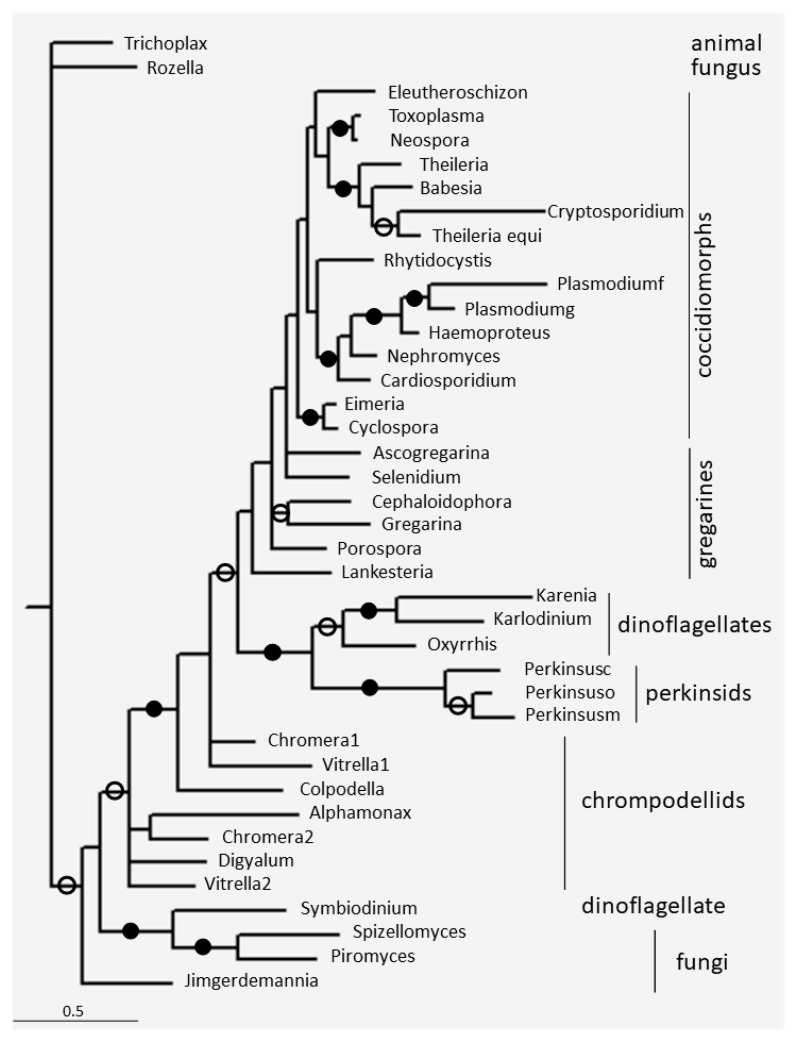
The phylogenetic tree of selected apicortins constructed by Bayesian analysis [25] using the WAG model [26]. The number of generations was 2.4 × 10^−6^. Filled and open circles at a node indicate that the branch was supported by the maximal Bayesian posterior probability (BPP) and ≥0.95 BPP, respectively. All the other branches were supported by BPP ≥ 0.5. The accession numbers of proteins are listed in Table 1 and Table S1.

The phylogenetic tree of apicortins was constructed (Figure 3). In addition to the apicortins of Myzozoa, an animal (Placozoa—*Trichoplax adhaerens*) and some fungal apicortins were also included. The apicortin tree mostly conforms to the known phylogenetic relationships (cf. Figure 2). Proteins of the Fungi/Metazoa group were separated from the myzozoan proteins; all the myzoan apicortins are within one clade except *Symbiodinium*. Coccidiomorphs are within one clade and are sisters to gregarines. Perkinsids are sisters to dinoflagellates. Apicomplexans (coccidiomorphs and gregarines) are sisters to (perkinsids and dinoflagelletes). All these groups together are sisters to chrompodellids. That is, the position of the chrompodellids and the (perkinsids and dinoflagellates) clade is reversed, as it should be according to the species phylogeny (cf. Figure 2). Within coccidiomorphs, Eimeriidae (*Eimeria* and *Cyclospora*) and Sarcocystidae (*Toxoplasma* and *Neurospora*) are separated from each other, which differs not only from the species phylogeny, but also from previous apicortin family trees [30,31].

### 4.2. Short-Type TPPP

**Figure 4:**
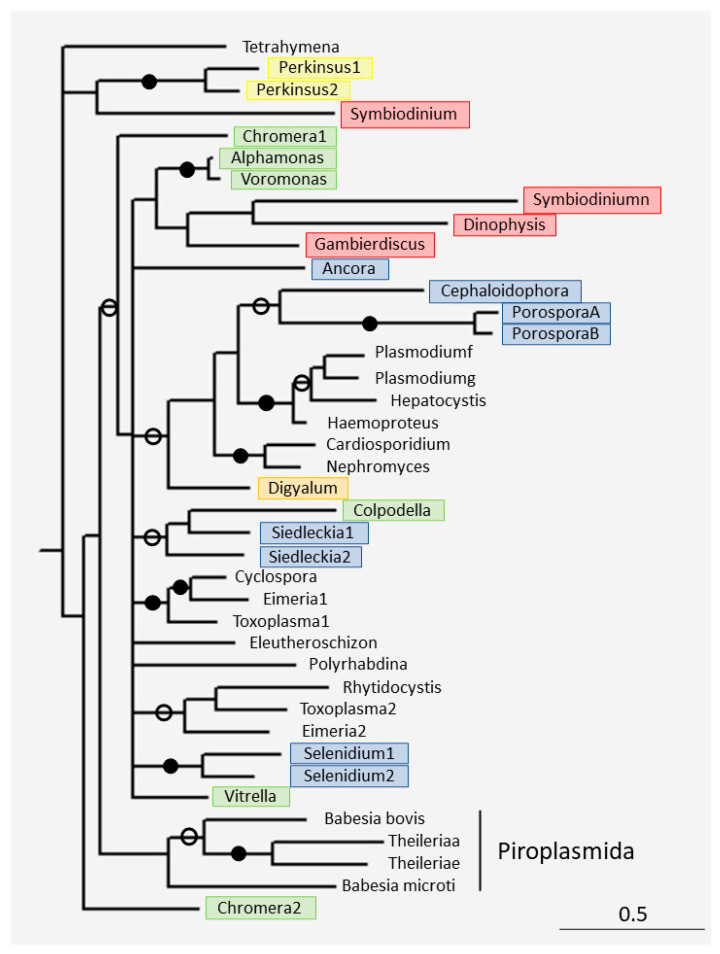
The phylogenetic tree of some short-type TPPPs was constructed by Bayesian analysis [25] using the GTR model [27]. The number of generations was 4 × 10^−6^. Filled and open circles at a node indicate that the branch was supported by the maximal Bayesian posterior probability (BPP) and ≥0.95 BPP, respectively. All the other branches were supported by BPP ≥ 0.5. The accession numbers of proteins are listed in Table 2 and Table S1. Color code: yellow—perkinsids; red—dinoflagellate; green—chrompodellids; orange—squirmids; blue—gregarines; no color—coccidiomorphs. *Tetrahymena thermophila* XP_977237 was used as outgroup.

The phylogenetic tree of short-type TPPPs was constructed (Figure 4). Many of the species contain more than one short-type TPPP. In several cases, the paralogs were included in the analysis. In some of the cases, gene duplication obviously occurred within the species (*Porospora*, *Siedlecka*, *Selenidium*, and *Perkinsus*), while in other cases, the duplication preceded the species speciation (*Toxoplasma*, *Eimeria*, and *Chromera*). Thus, in the first case, we can talk about inparalogs, while in the second case, we can talk of outparalogs [47].

The tree is not well-resolved and does not accurately reflect known phylogeny. Perkinsids and dinoflagellates form separate clades but chrompodellids and apicomplexans do not. Within Apicomplexa, gregarines, and coccidiomorphs are not well separated either. The most surprising thing is that the TPPPs of the Piroplasmida (*Babesia* and *Theileria*) order are not within the Apicomplexa, but in a sister position to them (for the discussion, see Section 5.3).

## 5. Possible Function of TPPP-like Proteins in Myzozoa

### 5.1. Tubulin-Based Structural Elements of Apicomplexa and Other Myzozoa

It is known that TPPP binds to tubulin, promotes its polymerization into microtubules, and stabilizes them [2,3]. This function is conserved in animals from sponges to mammals [12]. Tubulin-based cytoskeletal elements of apicomplexans have distinct traits, which may be related to the unique biology of these parasites [48]. In principle, TPPP-like proteins can interact with and stabilize any of these microtubular elements, which has been confirmed experimentally (cf. Section 5.2 and Section 5.3). The purpose of this article is not to provide a detailed overview of apicomplexan cytoskeleton; previous and more recent excellent reviews have done so [48,49,50].

Apicomplexans and their relatives possess discrete populations of microtubules and other tubulin polymers. Subpellicular microtubules and spindle microtubules are associated with the apical polar ring and with centrioles as the microtubule-organizing center, respectively. Axonemal microtubules are present in species possessing flagella in any of their life stages. Beside microtubules, some myzozoan species possess another polymer form of tubulin, the conoid fibers. They are similar to microtubules but their subunits are curled into an extremely tight coil, where tubulin is arranged into a polymer form that is different from typical microtubules [49]. Both the apical polar ring and the conoid are components of the namesake of apicomplexans, the apical complex, which was originally used for feeding in the common ancestor of apicomplexans and other myzozoans but was transformed for infection when the parasitism evolved [21,49].

It should be noted that the apical complex is complete only in some apicomplexans (e.g., Eimeriidae, Sarcocystidae, Cryptosporidiidae, and gregarines) [49]; other species of this phylum (in Piroplasmida and Haemosporida) lost the conoid, but they retained an apical polar ring; moreover, a conoid-like structure was found in the ookinete stage of *Plasmodium gallinaceum* [51] and in another haemosporidian, *Leucocytozoon simondi* [52]. Chrompodellids, but not *V. brassicaformis* [53], and perkinsids have an uncomplete ’pseudoconoid’, while dinoflagellates lack it but instead, they have another specialized tubular structure called peduncle [54].

### 5.2. Connection between Apical Complex/Conoid and Apicortin

The apical complex can be divided into three components, namely, the apical cap, the conoid, and the secretory organelles, micronemes, and rhoptries [49]. The conoid, which plays an important role in the host cell invasion, is best described from the apicomplexan families Eimeriidae and Sarcocystidae (*Sarcocystis*, *Toxoplasma*, *Neospora*, etc.). Apicortin seems to be in strict relation with this organelle. In *T. gondii*, apicortin was shown to be localized exclusively at the conoid and is essential for providing its correct structure and function [14,55]. Tubulin disappeared or was present in a reduced amount in the apical complex of an apicortin-null mutant, while microtubules were normal [55]. The deletion of apicortin resulted in shorter and misshaped conoids [55]. These defects were reversed by the expression of the apicortin coding sequence. Apicortin possesses two microtubule-binding domains (a partial p25alpha and a DCX), which stabilize and bundle microtubules [2,3,28,29]. Thus, apicortin is an ideal protein for stabilizing the tubulin-based conoid fibers. This stabilizing effect of apicortin was shown in a reconstituted in vitro system as well [14].

In *P. falciparum*, which has no conoid, apicortin is localized at the apical end and may be involved in the formation of the apical complex [56]. In both *T. gondii* and *P. falciparum*, the downregulation of apicortin leads to impaired host cell invasion [14,15]. Interestingly, in mammalian, but not in avian, *Plasmodium* parasites, the p25alpha domain is degenerated, i.e., some otherwise conservative amino acids are missing. At this moment, it is not clear yet whether the presence of a conoid-like structure in the ookinete stage of the avian parasite *P. gallinaceum* [51] is in connection with these sequence differences, or whether it happened accidentally, and a similar structure will also be found in mammalian parasites in the future. However, this altered sequence does not affect the ability of *P. falciparum* apicortin to bind to tubulin; in silico docking showed that the amino acids of the p25alpha domain are involved in the binding [56].

### 5.3. Flagella and Short-Type TPPP

TPPP-like proteins occur predominantly in species that are flagellated. The eukaryotic flagellum is a microtubule-based organelle; thus, the connection is not surprising. Although the correlation between the incidence of flagellum and the p25alpha domain is strong [5,6], there is only some evidence for the functional relationship between TPPP-like proteins and the flagellum. They are related to short-type TPPPs. The connection was first shown in *Chlamydomonas reinhardtii*, a biflagellate green alga [57]. Its TPPP ortholog, FAP265 protein, can be found in the flagella, and is indispensable in their formation, as proven by using FAP265 null mutants [57]. Myzozoan species are flagellated; however, apicomplexans are in a special position in this respect [58]. The flagella present in the common ancestor of the Myzozoa (and Alveolata) were lost during evolution. In many parasitic apicomplexans, flagella are only found in male microgametes with exceptions, such as Piroplasmida and the *Cryptosporidium* genus, where no flagellum occurs. Flagellum formation in apicomplexans does not require the so-called intraflagellar transport proteins, but all elements of the flagellar apparatus assemble directly within the cytoplasm in a process termed exflagellation [59].

There are examples for the flagellum-connected role of short-type TPPPs in Apicomplexa. In *Plasmodium* genus, *P. falciparum* short-type TPPP (PFL1770c; XP_001350760) was shown to be essential for gametocytogenesis, using piggyBac transposon-mediated insertional mutagenesis, but the mutant failed to form mature gametocytes [60]. Recently, it has also been found that the TPPP (*Py*05543; EAA17578) is necessary for male gametocyte exflagellation in *Plasmodium yoelli* [61]. *Py*05543^−^ KO parasites, obtained by CRISPR/Cas9-mediated genome editing, were deficient in the exflagellation of male gametes by observing deficient exflagellation center formation [61].

In this respect, it is noteworthy that *Cryptosporidium* species, which lack flagella [62,63,64], do not have short-type TPPPs. This can be interpreted as meaning that the parasite does not need this protein. A similar loss was described in *Cryptosporidium* for δ- and ε-tubulin genes/proteins, which are marker proteins for the flagellum and basal bodies [50]. Piroplasmids also have no flagella, not even in microgametes [65]. Their short-type TPPPs are the shortest among all short-type TPPPs. The lengths of TPPP of *Theileria annulata*, *B. microti*, and *B. bovis* are 121, 123, and 124 amino acids, respectively. The length of most short-type TPPPs is approximately 140–150 amino acids. Furthermore, among all apicomplexan TPPPs, the piroplasmid TPPPs are the most divergent (Table 4).

For example, the percent identity between *B. microti* and B. *bovis* is 31.93%, and between *B. microti* and *Theileria orientalis* (same order but different families), it is 26.45%. For the comparison: between *Toxoplasma gondii* and *Cyclospora cayetanensis* (same suborder but different families), the percent identity is 71.33%; between *Eimera necatrix* and *C. cayetanensis* (same family) it is 72.03%; between *Haemoproteus columbae* and *P. gallinaceum* (same order but different families) it is 78.29%; between *V. brassicaformis* and *E. necatrix* (different phyla) it is 53.57%; between *V. brassicaformis* and *T. thermophila* (different phyla) it is 52.08%. The separation of piroplasmid TPPPs from all the other apicomplexan TPPPs on the phylogenetic tree (Figure 4) may also be related to their divergent evolution. With the loss of the flagellum, their function may have been lost, so they evolved faster, and perhaps acquired a new function. However, in the apicortin tree, piroplasmids are deep within the apicomplexans, as expected according to the species phylogeny.

### 5.4. Function of the p25alpha Domain-Containing Multidomain Proteins

We find very different proteins in this group. Their length varies between 232 and 2627 amino acids. Interestingly, there are two short-type p25alpha domains in all of them. Sometimes, they do not have another domain; this type of protein is not found in apicomplexans and colpodellids, but is present in chromerids, perkinsids, and dinoflagellates. There is such a protein also in *D. oweni*; this species was previously classified as an apicomplexan, but according to the latest phylogenetic classification, it belongs to a separate group, the squirmids, which are sisters to (apicomplexans and chrompodellids) [36,37]. The fact that *D. oweni* has this type of protein (two p25alpha domains and no other domains) fits into this picture; i.e., *D. oweni* is not an apicomplexan. The function of these proteins is unknown; it would be expected that, similar to short-type TPPPs and apicortins, they exert their effect by binding to a tubulin polymer.

Only in dinoflagellates can we find larger proteins consisting of 1–2 thousand amino acids that contain more than three domains, including catalytic ones. For these proteins, the additional domains determine the function, e.g., adenylate cyclase or methyltransferase activity. The role of the p25alpha domains is unknown; however, there are cases when the importance of the tubulin-binding ability is likely, e.g., in the alpha-tubulin suppressor domain-containing protein CAI3986388 (*Cladocopium goreaui*). In the narrow sense, I do not consider these proteins to be TPPP-like ones.

## 6. Evolutionary Considerations

Among the TPPP-like proteins, short-type TPPPs and apicortins occur in all myzozoan phyla. Both experimental results and phylogenetic considerations indicate that these tubulin-binding proteins play an important role in the structure and/or function of the flagellum and the apical complex/conoid, respectively. Moreover, an evolutionary connection between the flagellum and apical complex was proposed [49,50]. Recent results suggest that the conoid complex evolved from flagellar components [66,67] or the flagellar root apparatus [49,68]; that is, ‘the apical complex is the most conspicuously retained element of the associated flagellar root structures’ [49].

In light of this, it is not surprising that short TPPPs are also common in Ciliata, the sister phylum of Myzozoa, since their surface is covered by hundreds of cilia; they usually contain several paralogs of short-type TPPPs. However, they do not have apicortin, and they do not possess conoid or any similar structural elements. The short-type TPPP is also present in other protists (Euglenozoa and Chlorophyta), which are also flagellated. Myzozoans are flagellated, but in apicomplexans, only the male gametes have flagellum/flagella in some species. If not, then the loss of the flagellum resulted in either the loss of TPPPs (*Cryptosporidium*) or a degenerate protein significantly diverging from other TPPPs (Piroplasmida). The conoid is also absent from some apicomplexans (Piroplasmida and Haemosporida), but despite this, apicortin is present in almost all of them. There can be several reasons for this. On the one hand, conoid-like structures are also found in some of these species [51,52], and on the other hand, the apical polar ring is also present in these cases; furthermore, since these species once possessed a conoid, the apicortin remained as a relic. The only exception is *B. microti*, which has an extremely small genome [40] and lacks the ‘redundant’ protein.

We cannot ignore the fact that apicortin is also found in some Opisthokonta (flagellated fungi, the placozoan *T. adhaerens*) [6,69], which do not have a conoid or similar structure. These apicortins differ from apicomplexan orthologs in that, although they have a partial p25alpha domain and the entire DCX domain, the opisthokont apicortin lacks the long, disordered N-terminal region [6,44], which appears to be necessary for the formation of the proper conoid structure [14]. Their function is probably to stabilize tubulin polymers/microtubules in these species as well; this is also indicated by the fact that apicortin is the only TPPP-like protein in *T. adhaerens* and some flagellated fungi [6,7]. Since apicortin is present only in these few primitive opisthokonts apart from Myzozoa, it is possible that the opisthokonts acquired this protein from myzozoans by horizontal gene transfer. This is supported by the high degree of sequence identity and similarity between apicomplexan and chromerid apicortins on the one hand, and *Trichoplax* and fungal apicortins on the other, which can reach 53% and 67%, respectively, which is roughly the same value as the similarity of apicomplexan apicortins to each other (Table S2). The similarity exists not only between the domain sequences, but also in the interdomain linker. Since the last common ancestor of myzozoans and opisthokonts could be close to the first eukaryote, it is extremely unlikely that the gene remained so conserved for that much time, and horizontal gene transfer is the best explanation.

## 7. Conclusions

A common feature of TPPP-like proteins is that they contain one or more p25alpha domains [1]. They occur only in eukaryotes, and like their name indicates, they are tubulin-binding proteins [2,3]. This trait is conserved in animals, as it is present in everything from sponges to humans [13]. The phylogenetic distribution of TPPP-like proteins is widespread in protists as well, but not in plants [1]. The role of TPPP-like proteins may be quite different in protists than in humans. Short-type TPPPs and apicortins are found in Myzozoa, a major monophyletic group of Alveolata. Although there are few experimental examples so far, short-type TPPPs have a role in the flagellar structures, interacting with tubulin polymers, and perhaps play a role in the exflagellation of microgametes [60,61]. Apicortin may play a crucial role in the formation of the conoid/apical complex [14,15,55,56]. Thus, TPPP-like proteins may be potential therapeutic targets for the treatment of diseases such as malaria and toxoplasmosis [50].

## Figures and Tables

**Table 1:** Newly identified apicortins.

Species	Accesion Number (TSA)	Class	Order	Identity with *T. adhaerens* Apicortin ^3^, %
**Apicomplexans**				
*Haemoproteus columbae*	GGWD01002623	Aconoidasida	Haemosporida	41.21
*Cardiosporidium cionae*	KAF8822549 ^1^	Aconoidasida	Nephromycida	36.67
*Nephromyces* sp. ex *Molgula occidentalis*	GHIL01104982	Aconoidasida	Nephromycida	40.35
*Eleutheroschizon duboscqi*	GHVT01063535	Conoidasida	Protococcidiorida	40.57
*Ancora sagittata*	GHVO01023203 ^2^	Conoidasida	Eugregarinorida	36.25
*Cephaloidophora* cf. *communis*	GHVH01004777	Conoidasida	Eugregarinorida	40.00
*Lankesteria abbotti*	HBHB01002866 ^2^	Conoidasida	Eugregarinorida	33.82
*Porospora* cf. *gigantea*	KAH0481109 ^1^	Conoidasida	Eugregarinorida	39.52
*Siedleckia nematoides*	GHVV01274235 ^2^	Conoidasida	Eugregarinorida (Blastogregarinorida)	51.35
*Selenidium pygospionis*	GHVN01000425 ^2^	Conoidasida	Archigregarinorida	35.66
*Rhytidocystis* sp. ex *Travisia forbesii*	GHVS01047420 ^2^ GHVS01057697 ^2^	Marosporida	Agamococcidiorida	37.65 37.11
**Chrompodellids**				
*Alphamonas edax*	GDKI01002741			36.26
*Colpodella angusta*	GDKK01042800			40.88
**Squirmids.**				
*Digyalum oweni*	GHRU01063100			48.52
**Dinoflagellates**				
*Karenia papilionacea*	GJRB01069842 ^2^ GFLM01035203 ^2^	Dinophyceae	Gymnodiniales	21.86 24.40
*Karlodinium armiger*	GJRA01036780 GJQZ01074857	Dinophyceae	Gymnodiniales	30.17 29.48
*Oxyrrhis marina*	HBQX01034077 HBIT01006257	Dinophyceae	Oxyrrhinales	33.33 33.33
*Symbiodinium* sp. clade D	HBTB01100466	Dinophyceae	Suessiales	38.31
**Perkinsids**				
*Perkinsus chesapeaki*	KAF4672084 ^1^		Perkinsida	23.90
*Perkinsus olseni*	KAF4750811 ^1^		Perkinsida	23.57

^1^ Protein. ^2^ Incomplete sequence.^3^ XP_002111209.

**Table 2:** Newly identified short-type TPPPs.

Species	Accesion Number (TSA)	Class	Order	Identity with *T. thermophila* TPPP ^5^, %
**Apicomplexans**				
*Haemoproteus columbae*	GGWD01012446+ GGWD01012443	Aconoidasida	Haemosporida	48.61
*Hepatocystis* sp. ex *Piliocolobus* *tephrosceles*	VWU48670 ^1^	Aconoidasida	Haemosporida	42.07
*Plasmodium gallinaceum*	XP_028529806 ^1^	Aconoidasida	Haemosporida	44.83
*Cardiosporidium cionae*	KAF8819752 ^1^	Aconoidasida	Nephromycida	50.68
*Nephromyces* sp. ex *Molgula occidentalis*	GHIL01028850	Aconoidasida	Nephromycida	49.32
*Babesia microti*	XP_012649535 ^1^	Aconoidasida	Piroplasmida	27.05
*Cyclospora cayetanensis*	XP_022592352 ^1^	Conoidasida	Eucoccidiorida	56.55
*Neospora caninum*	XP_003880535 ^1^ XP_003883867 ^1^	Conoidasida	Eucoccidiorida	56.94 50.34
*Eleutheroschizon duboscqi*	GHVT01063834	Conoidasida	Protococcidiorida;	55.56
*Ancora sagittata*	GHVO01049457	Conoidasida	Eugregarinorida	3730
*Cephaloidophora* cf. *communis*	GHVH01010051	Conoidasida	Eugregarinorida	44.59
*Gregarina niphandrodes*	GNI_040770 ^2^	Conoidasida	Eugregarinorida	37.04
*Polyrhabdina* sp.	GHVP01031609 ^3^	Conoidasida	Eugregarinorida	35.64
*Porospora* cf. *gigantea*	KAH0473834 KAH0475585	Conoidasida	Eugregarinorida	34.01 36.05
*Siedleckia nematoides*	GHVU01040477 GHVV01097121	Conoidasida	Eugregarinorida (Blastogregarinorida)	52.03 48.85
*Selenidium pygospionis*	GHVN01019431 ^4^	Conoidasida	Archigregarinorida	47.30, 45.64
*Rhytidocystis* sp. ex *Travisia forbesii*	GHVS01062019 GHVS01062016	Marosporida	Agamococcidiorida	41.22 43.24
**Chrompodellids**				
*Chromera velia*	HBKZ01015069 HBKZ01021816			61.38 40.69
*Vitrella brassicaformis*	CEM02660 ^1^			50.99
*Alphamonas edax*	GDKI01002338			56.16
*Colpodella angusta*	GDKK01046869			45.77
*Voromonas pontica*	GDKH01013421 ^3^			57.69
**Squirmids**				
*Digyalum oweni*	GHRU01021184			45.27
**Dinoflagellates**				
*Karenia mikimotoi*	GHKS01124154	Dinophyceae	Gymnodiniales	45.39
*Gambierdiscus australes*	HBLT01006012	Dinophyceae	Gonyaulacales	53.24
*Dinophysis acuminata*	GKBP01057714	Dinophyceae	Dinophysiales	30.07
*Symbiodinium* sp. *CCMP2456*	CAE7788281 ^1^	Dinophyceae	Suessiales	27.69
*Symbiodinium natans*	CAE7214312 ^1^	Dinophyceae	Suessiales	28.69
*Symbiodinium microadriaticum*	CAE7469644 ^1^	Dinophyceae	Suessiales	27.69
**Perkinsids**				
*Perkinsus marinus*	XP_002767104 ^1^		Perkinsida	32.47

^1^ Protein. ^2^ From https://cryptodb.org (accessed on 12 April 2023) [46]. ^3^ Incomplete sequence. ^4^ This TSA can be translated into two proteins. ^5^ XP_001023601.

**Table 3:** Newly identified multidomain proteins containing short p25alpha domains.

Species	Accession Number (Protein)	p25alpha Domains	Other Domains	Length (aa)
**Chrompodellids**				
*Chromera velia*	Cvel_4181 ^1,12^	2		232
	Cvel_31116 ^1^	2	EF-hand	499
*Vitrella brassicaformis*	CEL98751	2	EF-hand	433
**Squirmids**				
*Digyalum oweni*	GHRU01002363 ^2^	2		399
**Dinoflagellates**				
*Polarella glacialis*	CAE8635994	2	EF-hand	344
	CAE8640696	2	EF-hand	347
	CAE8623567	2	EF-hand	637
*Cladocopium goreaui*	CAI4012958	2	Nc ^3^ and others ^4^	1808
	CAI4010985	2	PRK08691 ^5^	1704
	CAI3986388	2	several ^6^	1281
*Symbiodinium* sp. CCMP2592	CAE7826117	2	sec 7 ^7^; Ank_2 ^8^, Nc and others ^9^	2611
	CAE7226582	2		333
*Symbiodinium* sp. CCMP2456	CAE7634140	2	sec 7; Nc	1368
	CAE7264691	2		333
*Symbiodinium* sp. *KB8*	CAE7914847		sec 7; Ank_2, Nc, and others ^10^	2627
	CAE7947347	2	PTZ00121 ^11^	2004
*Symbiodinium natans*	CAE7514938	2		338
	CAE7230103	2	Nc	735
*Symbiodinium microadriaticum*	CAE7819146		Nc	1367
	OLP89249	2	Nc	1405
	OLQ07037	2	PTZ00121	2338
	CAE7562082	2	PTZ00121	2161
*Symbiodinium necroappetens*	CAE7814517	2	sec 7; Nc	1356
	CAE7554473	2	PTZ00121	1082
*Symbiodinium pilosum*	CAE7666671	2	Nc	1354
**Perkinsids**				
*Perkinsus chesapeaki*	KAF4658947	2		349
*Perkinsus olseni*	KAF4688416	2		358
	KAF4711908	2	Ank_2	445
	KAF4678978	2	Ank_2	489

Identical colors indicate homologous (orthologous and paralogous) proteins. ^1^ From https://cryptodb.org [46]. ^2^ TSA. ^3^ Nc—Nucleotidyl_cyc_III = catalytic domains of the mononucleotidyl cyclases are part of the class III nucleotidyl cyclases. ^4^ PLN03218—maturation of RBCL 1. Predicted O-methyltransferase YrrM. ^5^ PRK08691—DNA polymerase III subunits gamma and tau. ^6^ DUF1415, DUF760 (unknown functions); ATS1—alpha-tubulin suppressor and related RCC1 domain. ^7^ Sec7 domain is a guanine nucleotide exchange factor. ^8^ Ank_2—ankyrin repeats. ^9^ Myosin and kinesin motor domain; DNA repair exonuclease SbcCD ATPase subunit. ^10^ Myosin and kinesin motor domain; chromosome segregation ATPase. ^11^ PTZ00121 is classified as a model that may span more than one domain. ^12^ In Cvel_4181, the N-terminal parts of its p25alpha domains, including the L(V)xxF(Y)xxF sequence, are missing.

**Table 4:** Pair-wise identities of several short-type TPPPs in percentages.

Species	*Babesia microti*	*Babesia bovis*	*Theileria orientalis*	*Toxoplasma gondii*	*Eimeria necatrix*	*Cyclospora cayetanensis*	*Haemoproteus columbae*	*Plasmodium gallinaceum*	*Vitrella brassicaformis*	*Tetrahymena thermophila*
*B. microti*	100	31.93	26.45	31.97	28.69	27.87	25.00	28.33	28.57	27.87
*B. bovis*	31.93	100	38.52	39.83	41.03	40.17	37.93	38.79	37.39	37.82
*T. orientalis*	26.45	38.52	100	36.59	35.26	36.07	37.70	35.25	34.96	36.22
*T. gondii*	31.97	39.83	36.59	100	62.94	71.33	47.14	43.57	53.19	55.56
*E. necatrix*	28.69	41.03	35.25	62.94	100	72.03	42.86	40.00	53.57	53.13
*C. cayetanensis*	27.87	40.17	36.07	71.33	72.03	100	50.00	45.00	56.43	55.94
*H. columbaes*	25.00	37.93	37.70	47.14	42.86	50.00	100	78.29	47.95	47.55
*P. gallinaceum*	28.33	38.79	35.25	43.57	40.00	45.00	78.29	100	44.52	43.36
*V. brassicaformis*	28.57	37.39	34.96	53.19	53.57	56.43	47.95	44.52	100	52.08
*T. thermophila*	27.87	37.82	36.22	55.56	53.15	55.94	47.55	43.36	52.08	100

The data were obtained by the Clustal Omega program [24]. Short-type TPPPs are listed in Table 2 and Table S1. Color code: gray—under 30%; yellow: 30–40%; blue: 50–60%; green: more than 60%.

## Data Availability

The data presented in this study are available in this paper and in the supplementary material.

## Supplementary Materials

The following supporting information can be downloaded at https://www.mdpi.com/article/10.3390/microorganisms11061528/s1; Table S1: Accession numbers of proteins shown in Figure 3 and Figure 4; Table S2: Pair-wise identities of several apicortins in percentages. Figure S1: Multiple alignment of newly identified short-type TPPP sequences.

## References

[1] Orosz F. (2012). A new protein superfamily: TPPP-like proteins. PLoS ONE.

[2] Hlavanda E., Kovács J., Oláh J., Orosz F., Medzihradszky K.F., Ovádi J. (2002). Brainspecific p25 protein binds to tubulin and microtubules and induces aberrant microtubule assemblies at substoichiometric concentrations. Biochemistry.

[3] Tirián L., Hlavanda E., Oláh J., Horváth I., Orosz F., Szabó B., Kovács J., Szabad J., Ovádi J. (2003). TPPP/p25 promotes tubulin assemblies and blocks mitotic spindle formation. Proc. Natl. Acad. Sci. USA.

[4] Takahashi M., Tomizawa K., Ishiguro K., Sato K., Omori A., Sato S., Shiratsuchi A., Uchida T., Imahori K. (1991). A novel brainspecific 25 kDa protein (p25) is phosphorylated by a Ser/Thr-Pro kinase (TPK II) from tau protein kinase fractions. FEBS Lett..

[5] Orosz F., Ovádi J. (2008). TPPP orthologs are ciliary proteins. FEBS Lett..

[6] Orosz F. (2021). On the TPPP-like proteins of flagellated Fungi. Fungal Biol..

[7] Orosz F. (2009). Apicortin, a unique protein, with a putative cytoskeletal role, shared only by apicomplexan parasites and the placozoan *Trichoplax adhaerens*. Infect. Genet. Evol..

[8] Lehotzky A., Tirián L., Tökési N., Lénárt P., Szabó B., Kovács J., Ovádi J. (2004). Dynamic targeting of microtubules by TPPP/p25 affects cell survival. J. Cell Sci..

[9] Lehotzky A., Lau P., Tokési N., Muja N., Hudson L.D., Ovádi J. (2010). Tubulin polymerization-promoting protein (TPPP/p25) is critical for oligodendrocyte differentiation. Glia.

[10] Kovács G.G., László L., Kovács J., Jensen P.H., Lindersson E., Botond G., Molnár T., Perczel A., Hudecz F., Mezo G. (2004). Natively unfolded tubulin polymerization promoting protein TPPP/p25 is a common marker of alpha-synucleinopathies. Neurobiol. Dis..

[11] Orosz F., Kovács G.G., Lehotzky A., Oláh J., Vincze O., Ovádi J. (2004). TPPP/p25: From unfolded protein to misfolding disease: Prediction and experiments. Biol. Cell.

[12] Ferreira N., Gram H., Sorrentino Z.A., Gregersen E., Schmidt S.I., Reimer L., Betzer C., Perez-Gozalbo C., Beltoja M., Nagaraj M. (2021). Multiple system atrophy-associated oligodendroglial protein p25α stimulates formation of novel α-synuclein strain with enhanced neurodegenerative potential. Acta Neuropathol..

[13] Oláh J., Szénási T., Szabó A., Kovács K., Lőw P., Štifanić M., Orosz F. (2017). Tubulin binding and polymerization promoting properties of Tubulin Polymerization Promoting Proteins are evolutionarily conserved. Biochemistry.

[14] Leung J.M., Nagayasu E., Hwang Y.C., Liu J., Pierce P.G., Phan I.Q., Prentice R.A., Murray J.M., Hu K. (2020). A doublecortin-domain protein of Toxoplasma and its orthologues bind to and modify the structure and organization of tubulin polymers. BMC Mol. Cell Biol..

[15] Chakrabarti M., Joshi M., Kumari G., Singh P., Shoaib R., Munjal A., Kumar V., Behl A., Abid M., Garg S. (2021). Interaction of Plasmodium falciparum apicortin with ?- and ?-tubulin is critical for parasite growth and survival. Sci. Rep..

[16] Orosz F. (2021). Truncated TPPP—An Endopterygota-specific protein. Heliyon.

[17] Gile G.H., Slamovits C.H. (2014). Transcriptomic analysis reveals evidence for a cryptic plastid in the colpodellid *Voromonas pontica* a close relative of chromerids and apicomplexan parasites. PLoS ONE.

[18] Janouškovec J., Tikhonenkov D.V., Burki F., Howe A.T., Kolísko M., Mylnikov A.P., Keeling P.J. (2015). Factors mediating plastid dependency and the origins of parasitism in apicomplexans and their close relatives. Proc. Natl. Acad. Sci. USA.

[19] Muñoz-Gómez S.A., Slamovits C.H. (2018). Plastid genomes in the Myzozoa. Adv. Bot. Res..

[20] Janouškovec J., Paskerova G.G., Miroliubova T.S., Mikhailov K.V., Birley T., Aleoshin V.V., Simdyanov T.G. (2019). Apicomplexan-like parasites are polyphyletic and widely but selectively dependent on cryptic plastid organelles. Elife.

[21] Wiser M.F. (2021). Unique Endomembrane Systems and Virulence in Pathogenic Protozoa. Life.

[22] Altschul S.F., Madden T.L., Schäffer A.A., Zhang J., Zhang Z., Miller W., Lipman D.J. (1997). Gapped BLAST and PSI-BLAST: A new generation of protein database search programs. Nucleic Acids Res..

[23] Amos B., Aurrecoechea C., Barba M., Barreto A., Basenko E.Y., Bażant W., Belnap R., Blevins A.S., Böhme U., Brestelli J. (2022). VEuPathDB: The eukaryotic pathogen, vector and host bioinformatics resource center. Nucleic Acids Res..

[24] Sievers F., Wilm A., Dineen D., Gibson T.J., Karplus K., Li W., Lopez R., McWilliam H., Remmert M., Söding J. (2011). Fast, scalable generation of high-quality protein multiple sequence alignments using Clustal Omega. Mol. Syst. Biol..

[25] Ronquist F., Huelsenbeck J.P. (2003). MrBayes 3: Bayesian phylogenetic inference under mixture models. Bioinformatics.

[26] Whelan S., Goldman N. (2001). A general empirical model of protein evolution derived from multiple protein families using a maximum-likelihood approach. Mol. Biol. Evol..

[27] Tavaré S., Miura R.M. (1986). Some probabilistic and statistical problems in the analysis of DNA sequences. Lect. Math. Life Sci..

[28] Sapir T., Horesh D., Caspi M., Atlas R., Burgess H.A., Wolf S.G., Francis F., Chelly J., Elbaum M., Pietrokovski S. (2000). Doublecortin mutations cluster in evolutionarily conserved functional domains. Hum. Mol. Genet..

[29] Kim M.H., Cierpicki T., Derewenda U., Krowarsch D., Feng Y., Devedjiev Y., Dauter Z., Walsh C.A., Otlewski J., Bushweller J.H. (2003). The DCX domain tandems of doublecortin and doublecortin-like kinase. Nat. Struct. Biol..

[30] Orosz F. (2022). On the TPPP protein of the enigmatic fungus, *Olpidium*—Correlation between the incidence of p25alpha domain and that of the eukaryotic flagellum. Int. J. Mol. Sci..

[31] Orosz F. (2016). Wider than thought phylogenetic occurrence of apicortin, a characteristic protein of apicomplexan parasites. J. Mol. Evol..

[32] Orosz F. (2023). Tubulin Polymerization Promoting Proteins (TPPPs) of Aphelidiomycota: Correlation between the incidence of p25alpha domain and the eukaryotic flagellum. J. Fungi.

[33] Moore R.B., Oborník M., Janouškovec J., Chrudimský T., Vancová M., Green D.H., Wright S.W., Davies N.W., Bolch C.J., Heimann K. (2008). A photosynthetic alveolate closely related to apicomplexan parasites. Nature.

[34] Oborník M., Modrý D., Lukeš M., Cernotíková-Stříbrná E., Cihlář J., Tesařová M., Kotabová E., Vancová M., Prášil O., Lukeš J. (2012). Morphology ultrastructure and life cycle of *Vitrella brassicaformis* n. sp., n. gen., a novel chromerid from the Great Barrier Reef. Protist.

[35] Woo Y.H., Ansari H., Otto T.D., Klinger C.M., Kolisko M., Michálek J., Saxena A., Shanmugam D., Tayyrov A., Veluchamy A. (2015). Chromerid genomes reveal the evolutionary path from photosynthetic algae to obligate intracellular parasites. Elife.

[36] Mathur V., Kwong W.K., Husnik F., Irwin N.A.T., Kristmundsson Á., Gestal C., Freeman M., Keeling P.J. (2021). Phylogenomics identifies a new major subgroup of apicomplexans, Marosporida *class nov*., with extreme apicoplast genome reduction. Genome Biol. Evol..

[37] Mathur V., Na I., Kwong W.K., Kolisko M., Keeling P.J. (2023). Reconstruction of plastid proteomes of apicomplexans and close relatives reveals the major evolutionary outcomes of cryptic plastids. Mol. Biol. Evol..

[38] Cavalier-Smith T. (2014). Gregarine site-heterogeneous 18S rDNA trees, revision of gregarine higher classification, and the evolutionary diversification of Sporozoa. Eur. J. Protistol..

[39] Koura E.A., Grahame J., Owen R.W., Kamel E.G. (1990). *Digyalum oweni*, *gen. nov*., *sp. nov*., a new and unusual gregarin protozoan from the gut of mollusc *Littorina obtusata* (Prosobranchia: Gastropoda). J. Egypt. Soc. Parasitol..

[40] Cornillot E., Hadj-Kaddour K., Dassouli A., Noel B., Ranwez V., Vacherie B., Augagneur Y., Brès V., Duclos A., Randazzo S. (2012). Sequencing of the smallest Apicomplexan genome from the human pathogen *Babesia microti*. Nucleic Acids Res..

[41] Boisard J., Duvernois-Berthet E., Duval L., Schrével J., Guillou L., Labat A., Le Panse S., Prensier G., Ponger L., Florent I. (2022). Marine gregarine genomes reveal the breadth of apicomplexan diversity with a partially conserved glideosome machinery. BMC Genom..

[42] Hunter E.S., Paight C., Lane C.E. (2020). Metabolic contributions of an alphaproteobacterial endosymbiont in the apicomplexan *Cardiosporidium ciona*. Front. Microbiol..

[43] Muñoz-Gómez S.A., Durnin K., Eme L., Paight C., Lane C.E., Saffo M.B., Slamovits C.H. (2019). *Nephromyces* represents a diverse and novel lineage of the Apicomplexa that has retained apicoplasts. Genome Biol. Evol..

[44] Orosz F. (2011). Apicomplexan apicortins possess a long disordered N-terminal extension. Infect. Genet. Evol..

[45] Bogema D.R., Yam J., Micallef M.L., Gholipourkanani H., Go J., Jenkins C., Dang C. (2021). Draft genomes of *Perkinsus olseni* and *Perkinsus chesapeaki* reveal polyploidy and regional differences in heterozygosity. Genomics.

[46] Warrenfeltz S., Kissinger J.C., EuPathDB Team (2020). Accessing Cryptosporidium omic and isolate data via CryptoDB.org. Methods Mol. Biol..

[47] Sonnhammer E.L., Koonin E.V. (2002). Orthology, paralogy and proposed classification for paralog subtypes. Trends Genet..

[48] Morrissette N.S., Sibley L.D. (2002). Cytoskeleton of apicomplexan parasites. Microbiol. Mol. Biol. Rev..

[49] Dos Santos Pacheco N., Tosetti N., Koreny L., Waller R.F., Soldati-Favre D. (2020). Evolution, composition, assembly, and function of the conoid in Apicomplexa. Trends Parasitol..

[50] Morrissette N.S., Abbaali I., Ramakrishnan C., Hehl A.B. (2023). The tubulin superfamily in apicomplexan parasites. Microorganisms.

[51] Patra K.P., Vinetz J.M. (2012). New ultrastructural analysis of the invasive apparatus of the *Plasmodium* ookinete. Am. J. Trop. Med. Hyg..

[52] Brockley Paterson W., Desser S.S. (1989). The polar ring complex in ookinetes of *Leucocytozoon simondi* (Apicomplexa: Haemosporida) and evidence for a conoid in haemosporidian ookinetes. Eur. J. Protistol..

[53] Füssy Z., Petra Masařová P., Kručinská J., Esson H.J., Oborník M. (2017). Budding of the alveolate alga Vitrella brassicaformis resembles sexual and asexual processes in apicomplexan parasite. Protist.

[54] Hansen P.J., Calado A.J. (1999). Phagotrophic mechanisms and prey selection in free-living dinoflagellates. J. Eukaryot. Microbiol..

[55] Nagayasu E., Hwang Y.C., Liu J., Murray J.M., Hu K. (2017). Loss of a doublecortin (DCX)-domain protein causes structural defects in a tubulin-based organelle of *Toxoplasma gondii* and impairs host-cell invasion. Mol. Biol. Cell.

[56] Chakrabarti M., Garg S., Rajagopal A., Pati S., Singh S. (2020). Targeted repression of *Plasmodium* apicortin by host microRNA impairs malaria parasite growth and invasion. Dis. Model. Mech..

[57] Tammana D., Tammana T.V.S. (2017). *Chlamydomonas* FAP265 is a tubulin polymerization promoting protein, essential for flagellar reassembly and hatching of daughter cells from the sporangium. PLoS ONE.

[58] Orosz F. (2021). Apicortin, a constituent of apicomplexan conoid/apical complex and its tentative role in pathogen-host interaction. Trop. Med. Infect. Dis..

[59] Avidor-Reiss T., Leroux M.R. (2015). Shared and distinct mechanisms of compartmentalized and cytosolic ciliogenesis. Curr. Biol..

[60] Ikadai H., Shaw Saliba K., Kanzok S.M., McLean K.J., Tanaka T.Q., Cao J., Williamson K.C., Jacobs-Lorena M. (2013). Transposon mutagenesis identifies genes essential for *Plasmodium falciparum* gametocytogenesis. Proc. Natl. Acad. Sci. USA.

[61] Zhang C., Li D., Meng Z., Zhou J., Min Z., Deng S., Shen J., Liu M. (2022). Pyp25α is required for male gametocyte exflagellation. Pathog. Dis..

[62] Ostrovska K., Paperna I. (1990). *Cryptosporidium* sp. of the starred lizard Agame stellio: Ultrastructure and life cycle. Parasitol. Res..

[63] Beĭer T.V., Sidorenko N.V. (1990). An electron microscopic study of Cryptosporidium. II. The stages of gametogenesis and sporogony in Cryptosporidium parvum. Tsitologiia.

[64] Tandel J., English E.D., Sateriale A., Gullicksrud J.A., Beiting D.P., Sullivan M.C., Pinkston B., Striepen B. (2019). Life cycle progression and sexual development of the apicomplexan parasite *Cryptosporidium parvum*. Nat. Microbiol..

[65] Adl S.M., Simpson A.G., Farmer M.A., Andersen R.A., Anderson O.R., Barta J.R., Bowser S.S., Brugerolle G., Fensome R.A., Fredericq S. (2005). The new higher level classification of eukaryotes with emphasis on the taxonomy of protists. J. Eukaryot. Microbiol..

[66] De Leon J.C., Scheumann N., Beatty W., Beck J.R., Tran J.Q., Yau C., Bradley P.J., Gull K., Wickstead B., Morrissette N.S. (2013). A SAS-6-like protein suggests that the *Toxoplasma* conoid complex evolved from flagellar components. Eukaryot. Cell.

[67] Portman N., Šlapeta J. (2014). The flagellar contribution to the apical complex: A new tool for the eukaryotic Swiss Army knife?. Trends Parasitol..

[68] Lévêque M.F., Berry L., Besteiro S. (2016). An evolutionarily conserved SSNA1/DIP13 homologue is a component of both basal and apical complexes of *Toxoplasma gondii*. Sci. Rep..

[69] Ringrose J.H., van den Toorn H.W.P., Eitel M., Post H., Neerincx P., Schierwater B., Altelaar A.F.M., Heck A.J.R. (2013). Deep proteome profiling of *Trichoplax adhaerens* reveals remarkable features at the origin of metazoan multicellularity. Nat. Commun..

