# Supplementary material for: p25alpha Domain-Containing Proteins of Apicomplexans and Related Taxa
Source: Microorganisms. 2023 Jun 8;11(6):1528. doi: 10.3390/microorganisms11061528 (PMC10304595; doi:10.3390/microorganisms11061528)
Supplement: Supplementary file 1 [file microorganisms-11-01528-s001.zip › microorganisms-2398778-supplementary.pdf]

**Table S1.** Accession Numbers of proteins shown in Figures 3 and 4[1]

| Name on the Figures | Species | Phylum | NCBI Accession No. |
|---|---|---|---|
| **Figure 3** | | | Apicortin |
| Trichoplax | *Trichoplax adhaerens* | Placozoa | XP_002111209 |
| Plasmodiumf | *Plasmodium falciparum* | Apicomplexa | XP_002808695 |
| Plasmodiumg | *Plasmodium gallinaceum* | Apicomplexa | XP_028530755 |
| Theileriaa | *Theileria annulata* | Apicomplexa | XP_952938 |
| Theileriaequi | *Theileria equi* | Apicomplexa | XP_004830792 |
| Babesia | *Babesia bovis* | Apicomplexa | XP_001609847 |
| Eimeria | *Eimeria necatrix* | Apicomplexa | XP_013434470 |
| Cyclospora | *Cyclospora cayetanensis* | Apicomplexa | XP_026194446 |
| Toxoplasma | *Toxoplasma gondii* | Apicomplexa | XP_002364910 |
| Neospora | *Neospora caninum* | Apicomplexa | XP_003883150 |
| Cryptosporidium | *Cryptosporidium parvum* | Apicomplexa | XP_001388280 |
| Gregarina | *Gregarina niphandrodes* | Apicomplexa | XP_011128898 |
| Ascogregarina | *Ascogregarina taiwanensis* | Apicomplexa | ABJQ010005682[2] |
| Perkinsusm | *Perkinsusm marinus* | Perkinsozoa | XP_006680205 |
| Chromera1 | *Chromera velia* | Chrompodellids | HBKZ01016309[3] |
| Chromera2 | | | JO810018[3] |
| Vitrella1 | *Vitrella brassicaformis* | Chrompodellids | CEM12737 |
| Vitrella2 | | | CEM06711 |
| Jimgerdemannia | *Jimgerdemannia flammicorona* | Mucoromycota | RUS30044 |
| Rozella | *Rozella allomycis* | Rozellomycota | EPZ32946 |
| Spizellomyces | *Spizellomyces punctatus* | Chytridiomycota | XP_016606225 |
| Piromyces | *Piromyces finnis* | Neocallimastigomycota | ORX59328 |
| **Figure 4** | | | Short-type TPPP |
| Tetrahymena | *Tetrahymena thermophila* | Ciliata | XP_977237 |
| Plasmodiumf | *Plasmodium falciparum* | Apicomplexa | XP_001350760 |
| Theileriaa | *Theileria annulata* | Apicomplexa | XP_953847 |
| Theileriaequi | *Theileria equi* | Apicomplexa | XP_004833523 |
| Babesia | *Babesia bovis* | Apicomplexa | XP_001610770 |
| Eimeria1 | *Eimeria necatrix* | Apicomplexa | XP_013438939 |
| Eimeria2 | | | XP_013439038 |
| Toxoplasma1 | *Toxoplasma gondii* | Apicomplexa | XP_002369913 |
| Toxoplasma2 | | | XP_002367917 |
| Perkinsus1 | *Perkinsus marinus* | Perkinsozoa | XP_002773110 |

[1]Accession Numbers listed in Table 1 and Table 2 are not shown here. [2]WGS. [3]TSA.

Table S2. Pair-wise identities of several apicortins in percentages

| | 1 Ta | 2 Jf | 3 Ra | 4 Pf | 5 S | 6 Do | 7 Cv | 8 Vb | 9 Pg | 10 Cp | 11 Ta | 12 Bb | 13 Pg | 14 En | 15 Tg |
|---|---|---|---|---|---|---|---|---|---|---|---|---|---|---|---|
| **METAZOA (Animals)** | | | | | | | | | | | | | | | |
| 1: Trichoplax | 100.00 | 51.61 | 52.83 | 38.51 | 37.75 | 50.00 | 48.73 | 46.84 | 40.65 | 39.46 | 45.81 | 40.00 | 35.26 | 40.00 | 40.65 |
| **FUNGI** | | | | | | | | | | | | | | | |
| 2: Jimgerdemannia | 51.61 | 100.00 | 51.46 | 35.81 | 38.51 | 45.16 | 53.21 | 48.10 | 45.16 | 40.82 | 45.16 | 49.68 | 37.18 | 43.87 | 44.52 |
| 3: Rozella | 52.83 | 51.46 | 100.00 | 30.00 | 38.83 | 43.40 | 40.57 | 44.34 | 42.86 | 40.95 | 39.05 | 39.05 | 38.10 | 38.10 | 36.19 |
| 4: Piromyces | 38.51 | 35.81 | 30.00 | 100.00 | 38.46 | 36.05 | 37.16 | 32.67 | 35.57 | 29.86 | 42.95 | 38.26 | 34.00 | 35.57 | 36.91 |
| **MYZOZOA** | | | | | | | | | | | | | | | |
| 5: Symbiodinium | 37.75 | 38.51 | 38.83 | 38.46 | 100.00 | 39.07 | 39.74 | 33.77 | 33.33 | 30.50 | 34.67 | 30.67 | 30.00 | 31.33 | 33.33 |
| 6: Digyalum | 50.00 | 45.16 | 43.40 | 36.05 | 39.07 | 100.00 | 51.90 | 55.06 | 44.52 | 37.41 | 46.45 | 42.58 | 40.38 | 43.23 | 44.52 |
| 7: Chromera | 48.73 | 53.21 | 40.57 | 37.16 | 39.74 | 51.90 | 100.00 | 57.86 | 44.87 | 39.86 | 42.31 | 44.23 | 41.40 | 43.59 | 41.03 |
| 8: Vitrella | 46.84 | 48.10 | 44.34 | 32.67 | 33.77 | 55.06 | 57.86 | 100.00 | 43.67 | 40.67 | 46.84 | 44.30 | 42.14 | 45.57 | 45.57 |
| 9: Porospora | 40.65 | 45.16 | 42.86 | 35.57 | 33.33 | 44.52 | 44.87 | 43.67 | **100.00** | **44.97** | **60.13** | **58.86** | **55.06** | **65.82** | **60.13** |
| 10: Cryptosporidium | 39.46 | 40.82 | 40.95 | 29.86 | 30.50 | 37.41 | 39.86 | 40.67 | **44.97** | **100.00** | **48.99** | **48.32** | **43.33** | **46.98** | **49.66** |
| 11: Theileria | 45.81 | 45.16 | 39.05 | 42.95 | 34.67 | 46.45 | 42.31 | 46.84 | **60.13** | **48.99** | **100.00** | **70.25** | **54.43** | **64.56** | **68.35** |
| 12: Babesia | 40.00 | 49.68 | 39.05 | 38.26 | 30.67 | 42.58 | 44.23 | 44.30 | **58.86** | **48.32** | **70.25** | **100.00** | **54.43** | **60.13** | **67.72** |
| 13: Plasmodiumg | 35.26 | 37.18 | 38.10 | 34.00 | 30.00 | 40.38 | 41.40 | 42.14 | **55.06** | **43.33** | **54.43** | **54.43** | **100.00** | **58.23** | **56.33** |
| 14: Eimeria | 40.00 | 43.87 | 38.10 | 35.57 | 31.33 | 43.23 | 43.59 | 45.57 | **65.82** | **46.98** | **64.56** | **60.13** | **58.23** | **100.00** | **74.05** |
| 15: Toxoplasma | 40.65 | 44.52 | 36.19 | 36.91 | 33.33 | 44.52 | 41.03 | 45.57 | **60.13** | **49.66** | **68.35** | **67.72** | **56.33** | **74.05** | **100.00** |

List of apicortins: Animal: *Trichoplax adhaerens*, XP_002111209; Fungi: *Jimgerdemannia flammicorona*, RUS30044; R*ozella allomycis*, EPZ32946; *Piromyces finnis*, ORX59328; Myzozoans: *Symbiodinium* sp. clade D, HBTB01100466; *Digyalum oweni*, GHRU01063100; *Chromera velia* JO810018; *Vitrella brassicaformis* CEM06711; *Porospora cf. gigantea* B, KAH0481109; *Cryptosporidium parvum*, XP_001388280; *Theileria annulate*, XP_952938; *Babesia bovis*, XP_001609847; *Plasmodiumg gallinaceum*, XP_028530755; *Eimeria necatrix*, XP_013434470; *Toxoplasma gondii*, XP_002364910. The N-terminal amino acids before the p25alpha domain were trimmed, i.e., the p25alpha and DCX domains and the interdomain linker region were used in the comparison. The data were obtained by the Clustal Omega program [24]. Color code: yellow: 45-50%; blue: 50-60%; green: more than 60%. Bold numbers indicate Apicomplexa – Apicomplexa pairs.

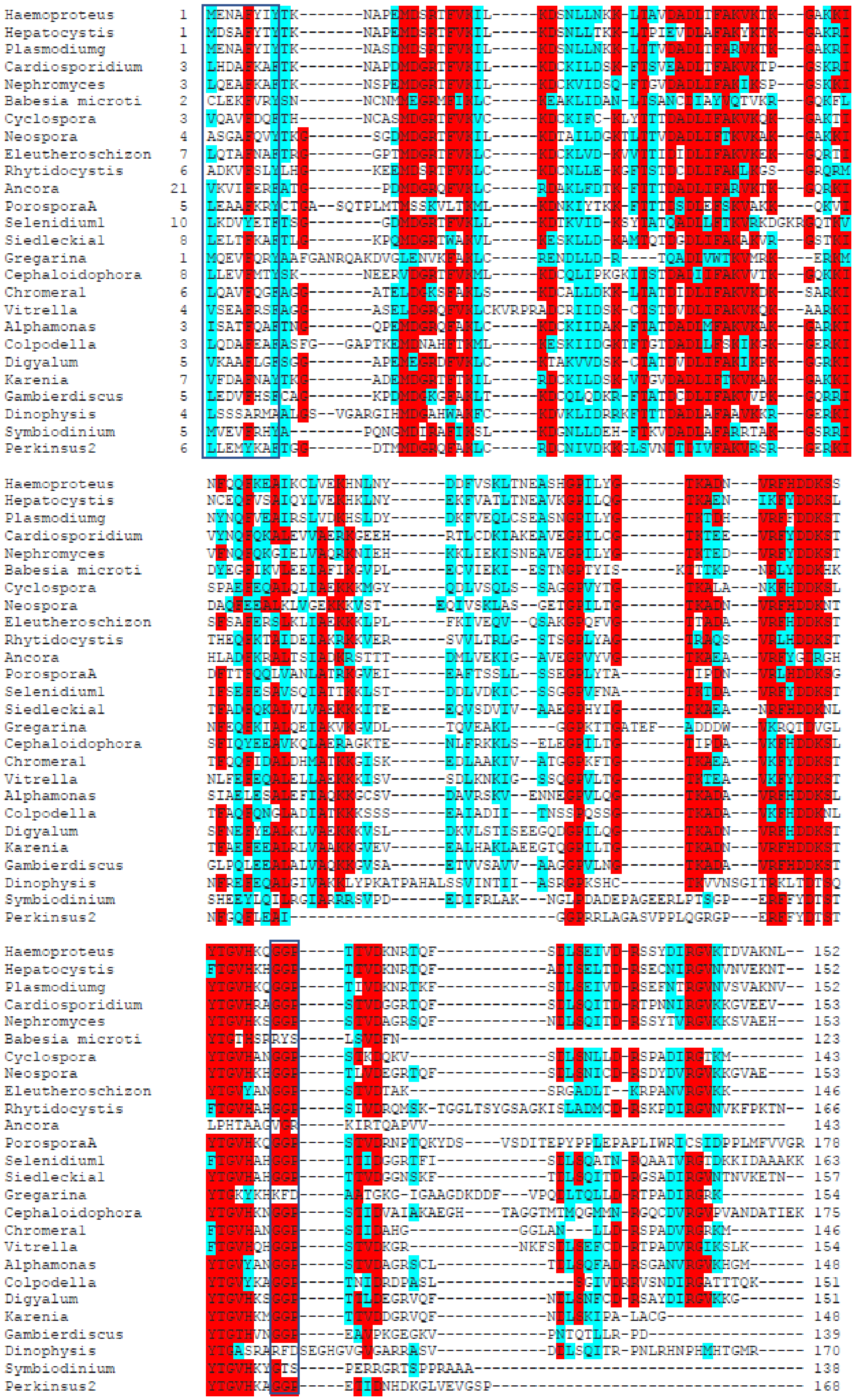

**Figure S1.** Multiple alignment of newly identified short-type TPPP sequences done by Clustal Omega [24] and refined manually. Only one fully sequenced protein/translated TSA was aligned per species. The NCBI Accession Numbers are given in Table 2. Identical and biochemically similar amino acids are labelled by red and blue background, respectively. The L(V)xxxF(Y)xxF and GGP conservative sequences are indicated with boxes.
